# Public Awareness of Rabies and Post-Bite Practices in Makkah Region of Saudi Arabia: Cross-Sectional Study

**DOI:** 10.3390/tropicalmed10120337

**Published:** 2025-11-29

**Authors:** Nahla H. Hariri, Khalid S. Alrougi, Abdullah A. Almogbil, Mona H. Kassar, Reman G. Alharbi, Abdullah O. Krenshi, Jory M. Altayyar, Abdullah S. Alibrahim, Maher N. Alandiyjany, Fozya B. Bashal, Nizar S. Bawahab, Saleh A. K. Saleh, Heba M. Adly

**Affiliations:** 1 Department of Community Medicine and Pilgrims Healthcare, College of Medicine, Umm Al-Qura University, Makkah 21955, Saudi Arabia; hmhasan@uqu.edu.sa; 2 College of Medicine, Umm Al-Qura University, Makkah 21955, Saudi Arabia; kalrwqy474@gmail.com (K.S.A.); almo8bail66@gmail.com (A.A.A.); monahkassar@gmail.com (M.H.K.); remanalh7@gmail.com (R.G.A.); abdullahkrenshi@gmail.com (A.O.K.); jorymaltayyar@gmail.com (J.M.A.); abdullahsal02a@gmail.com (A.S.A.); 3 Department of Clinical Laboratory Sciences, Faculty of Applied Medical Sciences, Umm Al-Qura University, Makkah 21955, Saudi Arabia; mnandiyjany@uqu.edu.sa; 4 Department of Internal Medicine, College of Medicine, Umm Al-Qura University, Makkah 21955, Saudi Arabia; fbbashal@uqu.edu.sa; 5 Department of General Surgery, King Faisal Hospital, Makkah 24236, Saudi Arabia; nbawahab@moh.gov.sa; 6 Ministry of Health Branch in the Makkah Region, Makkah 24231, Saudi Arabia; 7 Directorate of Institutional Excellence, Batterjee Medical College, Jeddah 21442, Saudi Arabia; saleh.abdrabuh@bmc.edu.sa

**Keywords:** rabies, public awareness, dog bites, post-exposure prophylaxis, Saudi Arabia, Makkah, One Health, Zero by 30

## Abstract

**Background:** Rabies is a fatal yet preventable zoonosis. In Saudi Arabia, uneven surveillance and limited public awareness may delay post-exposure prophylaxis (PEP). In Makkah, where residents regularly encounter free-roaming dogs, knowledge gaps could elevate exposure risks. **Objectives:** This study aims to assess public knowledge, attitudes, and post-bite practices regarding rabies, including wound washing and access to PEP among adult residents of the Makkah Region, and to examine associations with pet dog ownership. **Methods:** A cross-sectional survey was conducted in the Makkah Region (March–June 2025). An online validated bilingual questionnaire targeted residents ≥ 18 years via social media. Descriptive statistics, chi-square tests, 95% confidence intervals, and binomial logistic regression were applied in IBM SPSS v26; *p* < 0.05 was significant. **Results:** Of 523 respondents, 91.8% lived in Makkah city, 52.8% were female, and the age distribution was 18–24 years (44.2%), 25–34 years (35.6%), 35–44 years (12.0%), and ≥45 years (8.2%). Pet dog ownership was rare (1.9%), yet 39.4% reported stray dogs in their communities. Overall, 60.6% knew what rabies is and 63.7% knew it is vaccine-preventable, but 52.2% wrongly believed that transmission occurs only via dog bites. Hospitals (79.7%) and health centers (79.2%) were the most cited vaccination sites; social media was the dominant information source (74.6%). No significant association was found between pet ownership and rabies awareness (all *p* > 0.05). In multivariable regression (n = 509), adequate rabies knowledge increased the odds of an appropriate intended response (AOR 1.85, 95% CI: 1.27–2.68). Participants aged 30–40 years and those >50 years had significantly lower odds (AOR 0.45, 95% CI: 0.24–0.85 and AOR 0.23, 95% CI: 0.09–0.56, respectively). **Conclusions:** Despite moderate awareness, critical misconceptions and inconsistent first aid intentions persist. Priority actions include clear, locally adapted education on immediate wound washing and prompt PEP, standardized bite management pathways across facilities, reliable access to vaccines and immunoglobulin, and targeted social media micro-campaigns. By identifying public misconceptions, knowledge gaps, and preferred communication channels, this study provides baseline evidence to guide community awareness programs, intersectoral collaboration, and One Health-based surveillance essential for Saudi Arabia’s progress toward the global “Zero rabies by 2030” goal.

## 1. Introduction

Rabies remains a fatal yet fully preventable zoonosis that disproportionately affects countries in Asia and Africa. Human infection almost always follows an exposure to saliva from an infected animal typically through bites or scratches, with domestic dogs responsible for the vast majority of cases worldwide [1,2,3]. The causative agent is a neurotropic, single-stranded RNA virus of the genus Lyssavirus (family Rhabdoviridae) that enters peripheral nerves at the wound site and ascends to the central nervous system, producing rapidly progressive encephalitis [1,3]. Incubation is variable, ranging from weeks to >1 year, modulated by inoculum size, wound location, and host factors; early features often include low-grade fever and paresthesia or pain at the exposure site, followed by neurological manifestations that are almost invariably fatal without timely post-exposure prophylaxis (PEP) [1].

Despite the availability of effective vaccines and standardized PEP protocols, an estimated 59,000 people still die of rabies each year, largely among underserved rural populations with limited access to prompt wound care and biologics [1,2]. Global partners have therefore endorsed the “Zero by 30” target to end dog-mediated human rabies deaths by 2030 through coordinated mass dog vaccination, improved access to PEP, and strengthened surveillance [4,5,6]. Educational interventions, especially those aimed at school-aged children, who bear a disproportionate share of dog bite injuries, consistently improve knowledge and appropriate care-seeking after bites, supporting their inclusion alongside biomedical measures [7].

Within the Kingdom of Saudi Arabia (KSA), published data indicate that animal bites are a persistent public health concern and that rabies is enzootic among several species. National summaries from the late 2000s recorded more than eleven thousand animal bites to humans over three years, while veterinary and wildlife reports document positivity among dogs, foxes, camels, and other mammals [8]. Saudi data compiled by Al-Tayib et al. indicate that, among 40 laboratory-investigated animal rabies cases (2005–2010), 37 (92.5%) were confirmed positive, including 11 dogs, 6 foxes, 6 sheep, 5 camels, 4 goats, 3 wolves, and 2 cattle, underscoring multi-species transmission, with a notable canine contribution [9]. Recent regional syntheses further suggest gaps in standardized bite management pathways and in integrated human–animal surveillance, highlighting the need for contemporary, community-level data to guide awareness and prevention strategies. To date, no national estimate of dog rabies vaccination coverage has been published for Saudi Arabia; recent scoping reviews emphasize data gaps in vaccination metrics and One Health surveillance, while global guidance indicates that ≥70% dog vaccination coverage is needed to interrupt transmission [10,11].

This study aims to assesses public knowledge, attitudes, and practices related to rabies and dog bite management in the Makkah Region. By identifying misconceptions and behavioral barriers to prompt, appropriate PEP, the findings aim to inform locally tailored health education messages, strengthen referral pathways, and contribute evidence toward national efforts aligned with the “Zero by 30” framework.

## 2. Materials and Methods

### 2.1. Study Design, Setting

**Figure 1:**
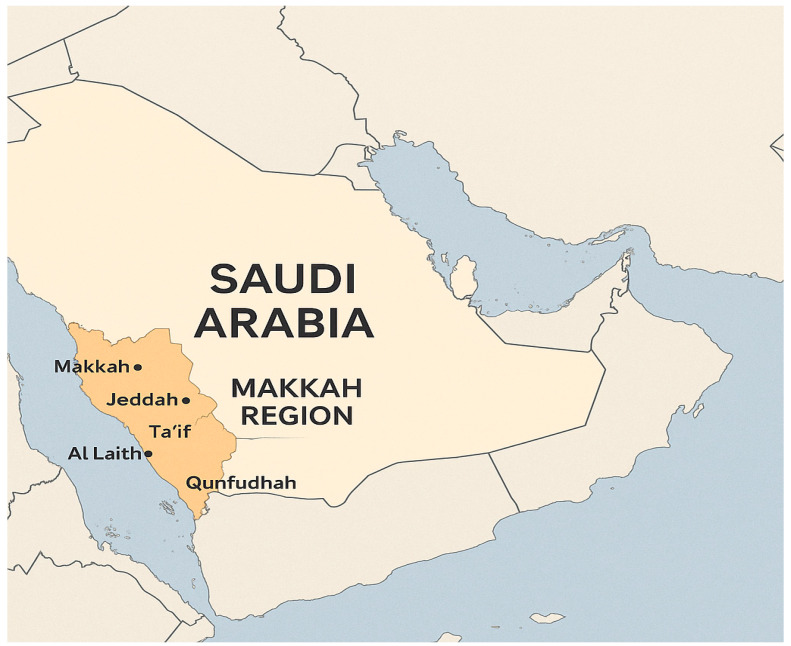
The Makkah Region is highlighted within the Kingdom of Saudi Arabia, showing the major cities included in the survey: Makkah, Jeddah, Ta’if, Al Laith, and Qunfudhah. Adopted from Wikipedia contributors. Saudi Arabia–Makkah region map (SVG). Wikimedia Commons; 2023.

The study was conducted in the Makkah Region, Saudi Arabia, including the cities of Makkah, Jeddah, Ta’if, Al Laith, and Qunfudhah (Figure 1). In this region, post-exposure prophylaxis (PEP) for rabies is administered within Ministry of Health services, primarily at public hospitals and primary healthcare centers.

### 2.2. Study Participants

The target population was adult residents (≥18 years) of the Makkah Region who provided electronic consent and completed an online questionnaire between March and June 2025. Recruitment focused on community residents to capture population-level awareness and intended care-seeking for bite management (including wound washing and access to PEP). Individuals working as healthcare providers and anyone declining participation were excluded.

### 2.3. Data Collection and Instrument

Data were collected using a self-administered online questionnaire (Google Forms) distributed via social media platforms targeting adult residents of the Makkah Region. The instrument comprised five sections adapted from prior work [12]: demographics: age, nationality, gender, city of residence, education level, and whether a family member/close friend works in the medical field; pet dog ownership: number, sex, age, breed, source, and reason for owning a dog; community dogs and attitudes/practices: care practices for community dogs (e.g., feeding, sterilization); dog bite and rabies awareness: bite prevention, rabies knowledge, and recommended responses to dog bites; link between pet ownership and rabies awareness/response: knowledge of screening/vaccination, perceived infection risk after a bite, vaccine effectiveness, and facilities to seek after exposure.

Scoring of knowledge: Each knowledge item was scored “1” for a correct response and “0” for an incorrect or “don’t know” response. The knowledge domain comprised five items, giving a theoretical score range of 0–5; total knowledge scores were converted to percentages as (observed score ÷ 5) × 100. Aggregate knowledge was classified as follows: Poor: 0–50% of total attainable score; Average: 51–75%; Good: >75%.

#### 2.3.1. Instrument Development, Translation, and Pilot Testing

Items were compiled from the published literature [12] by Ghosh et al., 2016, and underwent forward–backward translation. A medical expert and a bilingual (Arabic/English) translator independently translated the questionnaire into Arabic. Two additional bilingual experts blinded to the original back-translated the Arabic version into English. The back-translation was compared with the source to resolve discrepancies, following recommended WHO procedures [13,14].

The questionnaire was pre-tested on a group of participants of 20 adult residents from the Makkah Region to assess clarity, wording, and technical functionality. Minor adjustments to item phrasing and skip-logic were made based on their feedback before launching the final survey. Three subject matter experts evaluated content validity on a 4-point relevance scale (1 = insufficient/may remove; 2 = requires major revision; 3 = adequate/minor revision; 4 = sufficient). The content validity index (CVI)—the proportion of items rated 3 or 4—was 88.6%, indicating strong content validity. Internal consistency reliability was acceptable (Cronbach’s α = 0.78), exceeding the commonly used threshold of 0.70 [15].

#### 2.3.2. Ethics and Consent

The first survey page described study aims, procedures, voluntariness, and confidentiality. Proceeding with the questionnaire constituted informed consent. Ethical approval was obtained from the Institutional Review Board of Umm Al-Qura University (UQU), Makkah, Saudi Arabia (Approval No. HAPO-02-K-012-2025-03-2623).

#### 2.3.3. Data Management and Analysis

Sample size: The required sample size for this cross-sectional survey was calculated using the single-proportion formula:

n = Z^2^*p*(1 − *p*)/d^2^,

assuming a large source population of adult residents in the Makkah Region (total regional population ~ 7.8 million in 2022). With no prior local estimate of adequate rabies knowledge, we used a conservative expected proportion of 50% (*p* = 0.50), a 95% confidence level (Z = 1.96), and a margin of error of 5% (d = 0.05), which yielded a minimum required sample of 384 participants. To allow for approximately 20% non-response or incomplete questionnaires, we increased the target sample to about 460 participants. In practice, 523 adults completed the questionnaire, exceeding the minimum requirement and increasing the precision of the estimates.

All data were collected anonymously and handled in accordance with the ethical approval granted by the Umm Al-Qura University Research Ethics Committee. No identifying information (such as names, contact details) was collected at any stage. Each completed questionnaire was automatically assigned a non-identifiable numeric code to ensure confidentiality during data cleaning and analysis. Access to the raw dataset was restricted to the principal investigator and data analysts listed in the protocol, and the dataset was stored on a secure, password-protected server. All analyses were conducted on de-identified data, and results are reported only in aggregate form, ensuring that no individual participant can be identified.

Data were exported from Google Forms into IBM SPSS Statistics version 26 for analysis. Categorical variables were summarized as frequencies and percentages; continuous variables were summarized as mean ± standard deviation. Associations between participant characteristics and rabies knowledge categories were examined using Pearson’s chi-square (χ^2^) test. A two-sided *p*-value < 0.05 was considered statistically significant.

A binomial logistic regression model was fitted to identify factors associated with an appropriate intended response to a dog bite, defined as selecting both washing the wound and visiting a clinic/hospital to receive a rabies vaccine and not selecting doing nothing on the post-bite action item. The analysis was restricted to adult residents of the Makkah Region with complete data (n = 509). A composite rabies knowledge score was constructed from five factual items (knowing what rabies is, recognizing that a dog bite is not the only possible route, awareness that rabies is vaccine-preventable, awareness that animals can die from rabies, and knowing where to obtain a rabies vaccine); each correct answer was scored “1” and incorrect/don’t know was scored “0”. Scores were converted to percentages, and adequate knowledge was defined as ≥70% of the maximum score (≥4 of 5 items). Predictor variables entered into the model included gender, age group (18–29, 30–40, >50 years), household size (<3, 3–5, >5 members), prior dog bite history, attitudes toward dogs (agreement that dogs should be kept outside; agreement that dog numbers increase if not killed), and adequate knowledge status. Adjusted odds ratios (AORs) with 95% confidence intervals (CIs) were reported; statistical significance was set at *p* < 0.05.

## 3. Results

### 3.1. Participant Characteristics

A total of 523 respondents completed the questionnaire. Most were residents of the Makkah Region (509/523, 97.3%), of whom 91.8% (480/523) lived in the Makkah Region. Females constituted 52.8% (276/523) of the sample; 64.4% (337/523) reported households with >5 members (Table 1).

### 3.2. Pet Dog Ownership

Overall, 1.9% (10/523) reported owning pet dogs (Table 2A). Among these few owners, most kept a small number of adults, predominantly male dogs, and were often unsure of the breed. Dogs were usually obtained from previous litters, commonly kept for guarding, and were sometimes sterilized but frequently not officially registered (Table 2B). These estimates for pet dog ownership and care should be interpreted cautiously, given the very small number of dog owners in the sample (n = 10).

### 3.3. Community Dog Contact and Perceived Stray Burden

Among dog owners, around two-thirds reported caring for or feeding community dogs (Table 3A). At the population level, most respondents perceived at least some stray dogs in their neighborhood, most commonly estimating 1–5 animals (Table 3B).

### 3.4. Dog Bite History and Immediate Response

Only 0.8% (4/523) reported a previous dog bite; the exact Clopper–Pearson 95% CI for bite prevalence was 0.2–2.0%. Among those bitten (n = 4), the attacking dog was a stray in 2 cases, the respondent’s own dog in 1 case, and another person’s dog in 1 case (Table 4). The reported severity was predominantly medium (3/4, 75%).

### 3.5. Awareness, Attitudes, and Post-Bite Actions

Overall, 60.6% (317/523, 95% CI: 56.3–64.8%) reported that they know what rabies is, and 63.7% (333/523, 95% CI: 59.5–67.8%) recognized that rabies is preventable by a vaccine (Table 5A). Regarding appropriate post-bite behavior, 94.3% (493/523) selected visiting a clinic/hospital to receive a rabies vaccination, and 60.4% (316/523) selected washing wounds; smaller proportions reported seeking traditional treatments (8.6%) or doing nothing (2.9%) (Table 5B). Attitudinally, 89.7% agreed that dogs should be kept outside and 50.7% agreed that the number of dogs would increase if not killed (Table 5A). The most cited information source was social media (390/523, 74.6%), followed by doctors (41.3%) and TV (18.0%) (Table 5C).

### 3.6. Association Between Pet Ownership and Awareness Indicators

Bivariate χ^2^ tests (Table 6) showed no statistically significant association between pet dog ownership (10/523) and (i) rabies awareness (*p* = 0.488), (ii) the belief that a dog bite is the only route (*p* = 0.076, trending but not significant at α = 0.05), (iii) the perception that human deaths are high (*p* = 0.946), or (iv) the belief that animals die from rabies (*p* = 0.230). Given the very low prevalence of ownership, these analyses are underpowered to detect anything other than large effects.

### 3.7. Predictors of Appropriate Intended Post-Bite Action

**Figure 2:**
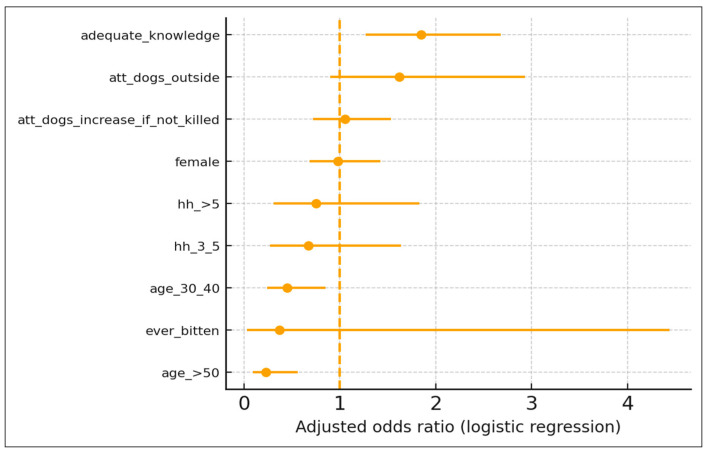
Predictors of appropriate intended rabies post-bite action among adults in the Makkah Region (n = 509). Adjusted odds ratios (AOR) and 95% confidence intervals derived from a binomial logistic regression model. An appropriate intended action was defined as washing the wound and visiting a clinic/hospital for rabies vaccination without choosing “do nothing.” Reference categories: male gender, age 18–29 years, household size < 3, inadequate knowledge, disagreement with “dogs should stay outside,” disagreement with “dog numbers increase if not killed,” and no previous dog bite.

Among 509 adult residents of the Makkah Region included in the regression analysis, 56.0% reported an appropriate intended response to a dog bite (washing the wound and attending a clinic/hospital for rabies vaccination without choosing to do nothing). In multivariable logistic regression, age and knowledge remained independently associated with the appropriate intended action (Figure 2). Compared with participants aged 18–29 years, those aged 30–40 years had significantly lower odds of an appropriate intended response (AOR 0.45, 95% CI: 0.24–0.85, *p* = 0.013), and those aged >50 years had even lower odds (AOR 0.23, 95% CI: 0.09–0.56, *p* = 0.001). Participants with adequate rabies knowledge (≥70% score) had a higher odds of intending to wash the wound and seek vaccination than those with a lower knowledge (AOR 1.85, 95% CI: 1.27–2.68, *p* = 0.001). Gender, household size, prior dog bite experience, and attitudes toward keeping dogs outside or killing dogs to control numbers were not significantly associated with the outcome (all *p* > 0.05).

## 4. Discussion

This community survey from the Makkah Region adds current evidence to public knowledge and bite response behaviors in a setting where dog ownership is uncommon but encounters with free-roaming dogs are reported. Although most respondents had never sustained a dog bite, self-reported knowledge was only moderate; about three in five said they “know what rabies is,” and a similar proportion recognized that rabies is vaccine-preventable, while over half believed (incorrectly) that infection occurs only via dog bites. These patterns align with the wider global picture: rabies remains a fully preventable zoonosis that still causes about 59,000 human deaths annually, largely in Asia and Africa, with dogs responsible for most human infections [16,17]. Despite the global Zero by 30 elimination agenda, uneven surveillance, access gaps to biologics, and limited community awareness continue to impede progress [18,19,20,21,22].

Two findings warrant an emphasis on program design. First, intended health-seeking after a bite was encouraging—nearly all respondents selected clinic/hospital vaccination, but wound washing (a critical first step) was selected far less frequently. This mirrors a well-described “knowledge–practice” gap in endemic settings and suggests that messaging should prioritize simple, actionable steps (immediate washing with soap and water for ≥15 min, then PEP) alongside destination guidance for vaccine/RIG [16,17,18]. Second, social media was the dominant information source; this creates both a vulnerability (misinformation) and an opportunity (precision public health campaigns). Evidence from recent syntheses shows that structured education, including school-based modules and community programs, improves knowledge and reported prevention practices and should be integrated with clinical pathways and animal health measures [23,24,25].

At the national and regional level, recent reviews highlight data gaps and the need for integrated, One Health-oriented bite management and surveillance across Saudi Arabia and the Arabian Peninsula [20,21,26]. Our null findings for associations between dog ownership and awareness are consistent with this systems-level picture: ownership per se may not drive knowledge where structured education and service access are variable. Priority actions consistent with Zero by 30 include (i) standardized triage and referral across hospitals/primary care sites; (ii) reliable availability of human vaccines and RIG; (iii) coordinated dog vaccination and responsible ownership policies; and (iv) targeted communication using the channels people actually use (here, social media), with rigorous monitoring and evaluation indicators [17,18,19].

This knowledge–practice gap may reflect several psychosocial and contextual factors documented in similar settings, including limited confidence in managing animal bites, misconceptions about wound washing, fear or uncertainty regarding the safety of rabies vaccines, and underestimation of the urgency of seeking PEP. In addition, the reliance on cultural beliefs or informal advice may discourage evidence-based first aid behaviors. Targeted health education, community-focused awareness campaigns, and clearer communication from front-line health services could strengthen self-efficacy, correct misconceptions, and improve timely first aid and PEP-seeking behaviors.

Strengths and limitations: This study uses a recent, region-specific sample to quantify awareness and post-bite intentions with clear denominators and CIs, providing a baseline for local programming. The limitations include the cross-sectional design, reliance on self-reporting, possible selection bias (online recruitment), and a very small number of dog owners and bite-exposed respondents, which limits the power for subgroup inference; however, these were minimized by using broad community-based recruitment across the entire Makkah Region, applying standardized and pre-tested survey questions, ensuring anonymous participation to reduce social desirability effects, and controlling for key socio-demographic and attitudinal variables through multivariable regression. Findings for very small subgroups (pet dog owners) are imprecise and should be interpreted with caution.

Future work should pair community surveys with facility-based audits of bite management (time to wound washing, PEP initiation, and completion) and animal health data on dog vaccination coverage, consistent with One Health monitoring recommended for Zero by 30 [17,18,19,21].

In practical terms, a locally adapted package that (i) standardizes first aid and PEP counseling at every point of care, (ii) scales school- and community-level education using validated curricula, and (iii) deploys social media micro-campaigns with bite response checklists could close the knowledge–practice gap quickly. These people-centered steps, combined with dog vaccination and surveillance investments, directly align with the current international guidance and Saudi/Gulf regional recommendations [16,17,18,19,20,21,24,25,26].

Within Saudi Arabia, official guidance recognizes rabies risk from dogs and some wildlife, with transmission prevention hinging on timely wound washing and PEP access [27]. Recent syntheses focused on the Arabian Gulf and the wider Arabian Peninsula highlight persistent surveillance and data gaps, uneven bite management pathways, and the need for integrated One Health coordination across human and animal sectors, priorities that map directly to the “Zero by 30” framework [28,29]. In practical terms, travelers’ health advisories and national clinical guidance converge on the point that human rabies vaccines are available across most of the country, but community awareness and standardized referral remain the limiting steps—underscoring the value of targeted, locally adapted education and reliable clinic pathways for vaccine/RIG [27,30]. A recent Saudi-focused narrative review reached a similar conclusion: strengthening bite management algorithms, public awareness, and intersectoral reporting are essential to close the knowledge–practice gap documented in local communities [31].

## 5. Conclusions

Despite a generally moderate awareness of rabies among Makkah residents, important misconceptions remain regarding the routes of transmission and appropriate preventive care. This study is one of the first community-based surveys in the Makkah Region to relate specific knowledge gaps and attitudes to intended post-bite practices, and it clearly demonstrates a gap between what people know and what they plan to do about wound care and seeking PEP. The findings point to the need for structured, locally adapted education campaigns that stress immediate wound cleansing and prompt access to PEP, with a particular attention to older adults and individuals with lower knowledge levels. As a practical next step, these messages should be incorporated into routine primary care, school-based activities, and wider community programs, and their impact monitored through repeated KAP surveys and behavioral indicators. Embedding such interventions within strengthened One Health surveillance and communication efforts will help support Saudi Arabia’s progress toward the global “Zero by 30” rabies elimination goal.

## Figures and Tables

**Table 1:** Demographic characteristics (n = 523).

Parameter	Category	n
Resident of Makkah Region	Yes	509 (97.3%)
	No	14 (2.7%)
Residence area	Makkah	480 (91.8%)
	Jeddah	29 (5.5%)
	Al Laith	1 (0.2%)
	Qunfudha	3 (0.6%)
	Ta’if	10 (1.9%)
Gender	Male	247 (47.2%)
	Female	276 (52.8)
Household size	<3	27 (5.2%)
	3–5	159 (30.4%)
	>5	337 (64.4%)

**Table 2:** (**A**). Pet dog ownership status (n = 523). (**B**). Characteristics among dog owners (n = 10).

**A**		
Parameter	Category	n
Pet dog ownership	Yes	10 (1.9%)
	No	513 (98.1%)
**B**		
Parameter	Category	n (% of owners)
Dogs cared for	<3	8 (80%)
	3–7	1 (10.0%)
	>7	1 (10.0%)
Dog sex	Male	6 (60.0%)
	Female	4 (40.0%)
Dog age	Puppy	2 (20.0%)
	Adult	8 (80.0%)
Breed	Unknown	5 (50.0%)
	Border collie	1 (10.0%)
	Chihuahua	0 (0.0%)
	Caucasian	2 (20.0%)
	Hybrid	2 (20.0%)
Source	Previous dog litter	9 (90.0%)
	Adoption from street	1 (10.0%)
Reason for ownership	Guarding (Yes)	8 (80.0%)
Sterilization	Yes	6 (60.0%)
	Maybe	4 (40.0%)
Official registration	Yes	3 (30.0%)
	No	7 (70.0%)

**Table 3:** (**A**). Caring/feeding community dogs among dog owners (n = 10). (**B**). Perceived stray dogs in the community (n = 523).

**A**			
Parameter	Category	n	% of owners
Ever cared for/fed community dogs	Yes	6	60.0
	No	4	40.0
**B**			
Category	n (%)		
None	137 (26.2%)		
I don’t know	111 (21.2%)		
1–5	206 (39.4%)		
6–10	40 (7.6%)		
11–20	10 (1.9%)		
>20	19 (3.6%)		

**Table 4:** Dog responsible for the bite (n = 4).

Attacking Dog	n (%)
Stray	2 (50.0%)
Own pet dog	1 (25.0%)
Other’s pet dog	1 (25.0%)

**Table 5:** (**A**). Awareness and attitudes (n = 523). (**B**). Post-bite actions (n = 523). (**C**). Knowledge of vaccine access points and information sources (n = 523).

**A**		
**Item**	**Category**	**n (%)**
Know what rabies is	Yes	317 (60.6%)
Rabies is vaccine-preventable	Yes	333 (63.5%)
Only way to get rabies is dog bite	Agree	273 (52.2%)
Dogs should be kept outside	Agree	469 (89.7%)
Dogs will increase if not killed	Agree	265 (50.7%)
**B**		
**Action**		**n (%)**
Visit clinic/hospital for rabies vaccine		493 (94.3%)
Wash wounds		316 (60.4%)
Search for traditional treatment		45 (8.6%)
I don’t know		29 (5.5%)
Do nothing		15 (2.9%)
Sterilize the area (antiseptic only)		1 (0.2%)
**C**		
**Item**	**Category**	**n**
Where vaccine is available	Hospital	417 (79.7%)
	Health center	414 (79.2%)
	Pharmacy	31 (5.9%)
Perceived severity	Human deaths from rabies are high (Agree)	162 (31.0%)
	Animals die from rabies (Agree)	306 (58.5%)
Information source	Social media	390 (74.6%)
	Doctors	216 (41.3%)
	TV	94 (18.0%)
	Other	21 (4.0%)
	General knowledge	1 (0.2%)

**Table 6:** Pet dog ownership vs. awareness outcomes (n = 523; owners n = 10).

Awareness Item	Ownership = No (n = 513), n (%)	Ownership = Yes, (n = 10) n (%)	*p*-Value
Know what rabies is (Yes vs. No) *	312 (60.8) vs. 201 (39.2)	5 (50.0) vs. 5 (50.0)	0.488
Only way is dog bite (Agree vs. Disagree)	265 (51.7) vs. 248 (48.3)	8 (80.0) vs. 2 (20.0)	0.076
Human deaths high (Agree vs. Disagree)	159 (31.0) vs. 354 (69.0)	3 (30.0) vs. 7 (70.0)	0.946
Animals die (Agree vs. Diagree)	302 (58.9) vs. 211 (41.1)	4 (40.0) vs. 6 (60.0)	0.230

* Row shows counts split by response (yes vs. no). Percentages are column-wise within ownership category.

## Data Availability

Data available on request.
